# Difference in whole spinal alignment between supine and standing positions in patients with adult spinal deformity using a new comparison method with slot-scanning three-dimensional X-ray imager and computed tomography through digital reconstructed radiography

**DOI:** 10.1186/s12891-018-2355-5

**Published:** 2018-12-06

**Authors:** Kazuhiro Hasegawa, Masashi Okamoto, Shun Hatsushikano, Gabriel Caseiro, Kei Watanabe

**Affiliations:** 1 Niigata Spine Surgery Center, 2-5-22 Nishi-machi, Niigata City, 950-0165 Japan; 2 EOS imaging, Paris, France; 3 Department of Orthopaedic Surgery, Niigata University Medical and Dental Hospital, Niigata, Japan

**Keywords:** Adult spinal deformity, Computed tomography, Digital reconstructed radiolography, Slot-scanning 3D X-ray imager (EOS), Supine and standing position, Whole spinal alignment

## Abstract

**Background:**

A precise comparison of supine and standing whole spine alignment in both the coronal and sagittal planes, including the pelvic parameters, has not been reported. Furthermore, previous studies investigated positional differences in the Cobb angle only in young patients with idiopathic scoliosis. The difference in alignment has never been investigated in a population of patients with adult spinal deformity (ASD). In most cases, ASD patients are aware of the symptoms when standing and tend to stoop with back pain, whereas the symptoms disappear when lying on a bed. Therefore, it is important to elucidate the positional differences in the deformity in older adults. The purposes of this study are to establish a method for comparing whole spine alignment between supine and standing, and to clarify the positional difference of the alignment in the patients with ASD.

**Methods:**

Twenty-four patients with ASD (mean age: 60.1 years, range 20–80 years; 24 women) were evaluated. A slot-scanning three-dimensional X-ray imager (EOS) was used to assess the whole spine in the standing position. Computed tomography was used to assess the whole spine in the supine position. The computed tomography DICOM dataset of the whole spine in the supine position was transformed to two-dimensional (coronal and sagittal) digital reconstructed radiography images. The digital reconstructed radiography images were input for three-dimensional measurement by the EOS software and compared with the standing whole spine alignment measured by EOS.

**Results:**

The mean intraclass correlation coefficients (supine, standing) of intra-rater / inter-rater reliabilities for the measured parameters were 0.981, 0.984 / 0.970, 0.986, respectively. The Cobb and rotation angles of the major curve, mostly the thoracolumbar area, were significantly greater in the standing position than in the supine position. Lumbar lordosis during standing was significantly kyphotic. With respect to the pelvic parameters, the sacral slope was significantly smaller in the standing position than in the supine position. Pelvic tilt and pelvic incidence were significantly greater in the standing position than in the supine position.

**Conclusions:**

The lumbar to pelvic parameters and the major curve in standing position significantly deteriorate compared with the supine position in patients with ASD.

## Background

Spinal deformity is a three-dimensional abnormality. Alignment is usually assessed in standing radiographs in both the sagittal and coronal planes using the Cobb angle [4]. When a patient presents with neurologic symptoms, details of the abnormality are usually assessed by referring to computed tomography (CT) images or magnetic resonance images (MRI) obtained with the patient in a supine position. Previous studies of adolescent idiopathic scoliosis (AIS) report major Cobb angles 7–10° smaller in the supine position than in the standing position due to changes in the gravitational loading direction [1, 21, 28, 30]. Therefore, to understand the pathology of the whole spine, it is important to determine the difference in the curve measurement between the supine and standing positions, including alignment. A drawback of previous reports is the potential error related to different measurement methods used for X-ray and other imaging modalities (CT or MRI) to compare the Cobb angle, which may lead to misinterpretation or controversy [1, 20, 21, 28, 30].

To our knowledge, a precise comparison of supine and standing measurements using the same modality and measurement method to overcome this drawback has not been reported. Whole spine alignment in both the coronal and sagittal planes, including the pelvic parameters, with regard to a positional difference has not been investigated. Furthermore, previous studies investigated positional differences in the Cobb angle only in young patients with AIS. The difference in alignment has never been investigated in a population of patients with adult spinal deformity (ASD). In contrast to AIS, the chief complaint of patients with ASD is usually mechanical back pain with or without radicular pain to the lower extremities. In most cases, the patients are aware of the symptoms when standing and tend to stoop with back pain, whereas the symptoms disappear when lying on a bed. Therefore, it is important to elucidate the positional differences in the deformity in older adults.

The purposes of this study were to: 1) Establish a method of comparing whole spine alignment between the supine and standing positions with minimal measurement error; and 2) Clarify the difference in the whole spine alignment between the supine position and the weight-bearing standing position in adult-to-elderly patients with spinal deformity.

## Methods

### Measurement of standing whole spine alignment with the EOS system

Comparison of whole spine alignment between the supine and standing positions necessitates high reproducibility of the imaging technology. Conventional X-ray measurement with a cone-beam X-ray provides significant magnification of the subject within the margin of the cassette. Therefore, we used the EOS system (EOS Imaging, Paris, France) as a principal device for measuring whole spine alignment [16]. The EOS system is a slot-scanning three-dimensional (3D) X-ray imager developed in collaboration by multidisciplinary partners, radiation physics engineers, biomechanical engineers, medical radiologists, and orthopedic pediatric surgeons to overcome the limitations of conventional X-ray measurement. From the simultaneous anteroposterior and lateral X-rays of the whole body to the 3D bone external envelope technique, 3D reconstruction is possible at every level of the osteoarticular system and especially the spine in the standing position. The EOS allows for more precise bone reconstruction in orthopedics, especially at the level of the spine, pelvis, and lower limbs, with limited X-ray exposure compared with conventional X-rays and CT scans [6, 7, 9, 19].

Radiographs with the EOS system [3, 9] were routinely obtained, as follows: 1) EOS radiographs were made from the head, including the center of the auditory canal, to the feet. 2) Each patient was asked to stand comfortably on a force plate while placing their hands on their cheeks. 3) A mirror placed at eye level in the inner wall of the EOS cabin helped the patient maintain a horizontal gaze [16].

The default scan speed of the EOS system was 7.6 cm/s. Acquisition time was linked to scan height: Time of acquisition (s) = height of acquisition (cm)/7.6. Scan speed can be increased if the patient is restless and having difficulty keeping still during the acquisition. Nevertheless, subtle artifacts in the images can occur due to body sway during scanning, but these are minimized because of the rapid X-ray detection time (0.8333 ms) with no blurring of the images. Some accessories are available to stabilize the patient in the EOS cabin. A recent study demonstrated that motion artifacts do not affect spinal measurements [26].

### Measurement of supine whole spine alignment with CT-generated digital reconstructed radiographs

**Fig. 1:**
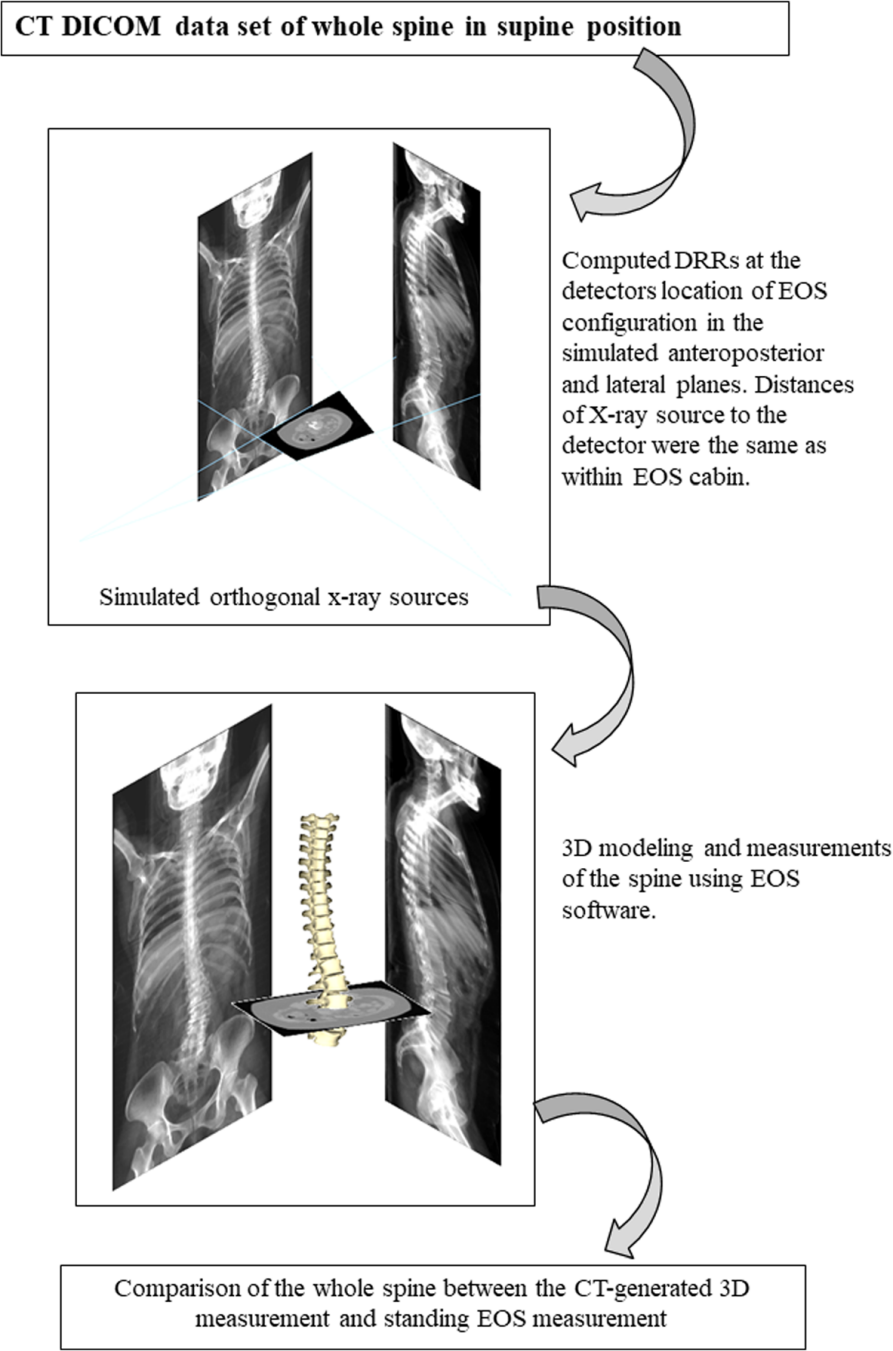
Conversion of supine CT data into digital reconstructed radiography (DRR) for comparable EOS measurement

**Fig. 2:**
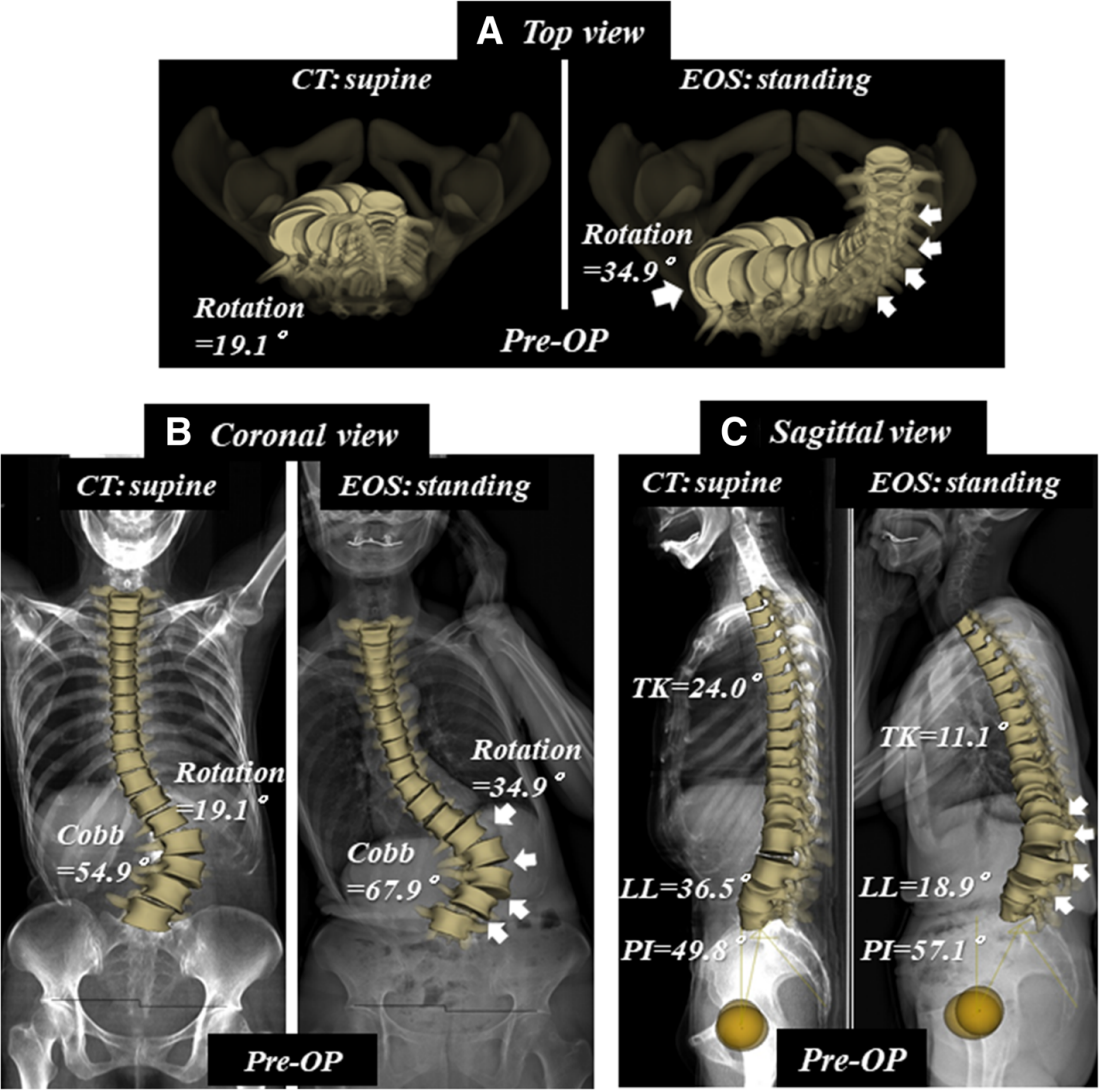
Patient with degenerative kyphoscoliosis. Preoperative supine CT and standing EOS images. **a** Top view images. Arrows show deterioration of T1 off-set and thoraco-lumbar rotation in standing position. **b** Coronal images. Arrows show deterioration of thoraco-lumbar curve in standing position. **c** Sagittal images. Arrows show deterioration of thoraco-lumbar kyphosis in standing position

Following EOS scanning, the whole spine, including the head and pelvis of the patient, was also scanned in the supine position with CT (Activion16, TSX-031A, Toshiba Medical Systems Corp., Tochigi, Japan). Currently, the most accurate 3D bone information may be obtained with CT imaging. To eliminate software-related bias and to guarantee equivalent computational methods during spinopelvic parameter comparisons between the supine position in the CT and the standing position in the EOS, the same analysis software must be used. Therefore, we chose to transform the CT dataset into an EOS-like dataset using the digitally reconstructed radiograph (DRR) technique. The DRR technique consists of simulating X-rays passing through the reconstructed CT volume based on an absorption-only optical model, thus generating an X-ray-like image. These biplanar projections were reconstructed using the same calibration parameters and geometry as the EOS cabin (Fig. 1). These projected anteroposterior and lateral DRRs, as well as those performed with the EOS system, were used as inputs for stereoradiographic spine modeling. Thus, for each position, 3D spine modeling was obtained using sterEOS software (sterEOS 1.6, EOS Imaging, Paris, France) [15] with both the EOS data (standing position) and the CT-generated DRRs (supine position), and compared between the two positions (Fig. 2).

### Spino-pelvic parameters for comparison

Using the full-spine workflow of the sterEOS software, the following spinopelvic parameters were calculated: kyphosis T1–T12 and T4–12, lumbar lordosis (LL) with respect to L1–L5 and L1–S1, pelvic tilt (PT), sacral slope (SS), and pelvic incidence (PI). PI is the angle between the line perpendicular to the sacral plate at its midpoint and the line connecting the midpoint of the sacral plate to the center of the axis connecting both acetabulae. PT is the angle defined between the line connecting the midpoint of the sacral plate to the center of the axis of both acetabulae and the vertical axis. SS is the angle between the sacral plate and the horizontal line. Regarding the deformity, the Cobb angle of the major curve (Cobb angle) and the axial vertebral rotation of the apex in the major curve (Rotation) were measured. All of the parameter results were compared between the supine (CT-generated DRR) and standing (EOS) positions.

### Clinical subjects

After obtaining institutional review board approval (Approval number 2, 27th Dec., 2013, Institutional Review Board of Kameda Daiichi Hospital, Niigata, Japan), we prospectively enrolled patients with ASD under the following criteria: 1) diagnosis: degenerative and idiopathic spinal kyphoscoliosis with Cobb angle more than 30° or degenerative kyphosis with PI-LL mismatch more than 20**°,** 2) age: > 20 years old, 3) sex: woman, 4) candidate for surgical treatment, 5) a full-spine CT scan (acquired from auditory canals to the proximal third of the femur) and a full-spine EOS image in both preoperative and postoperative states, 6) study term: from April 2014 to March 2016. The following demographic characteristics were obtained for each patient: age, sex, weight, and height. The body mass index was calculated as the weight in kilograms divided by the square of the height in meters. Patients with transitional vertebrae were excluded for precise measurement and comparison between supine and standing positions. Regarding the gender difference, we found a significant difference between men and women in PT, pelvic thickness, SVA, and lower extremity alignment in the previous study [17]. If men are included, the result may be affected. Therefore, men are excluded in this study. Consequently, a total of 24 cases with a mean age of 60.1 years (range: 20–80 years; 24 women) were analyzed after obtaining of informed consent for participation in the study. We used the Japanese version of the Oswestry Disability Index (ODI) [12, 13] and Scoliosis Research Society-22 score (SRS-22) [2, 18] to assess the health-related quality of life. ODI and SRS-22 are the principal condition-specific outcome measures used in the management of low back disorders and spinal deformities, respectively. Normal values without symptoms are 0 (%) in the ODI and 5 in the SRS-22, with the worst values being 100 (%) in the ODI and 0 in the SRS-22.

### Statistical analysis

JMP (version 9; SAS Institute, Cary, NC) and SPSS (IBM SPSS Statistics for Windows, Version 24.0, IBM Corp., Armonk, NY) were used for all statistical analyses. Mean, range, standard deviation (SD), standard error (SE), and the interquartile range, 25%/75%, were calculated for all the demographic and radiographic parameters. All variance-dependent variables were checked for normality and homogeneity of variance. Alpha was set at *p* < 0.05.

An intra-class correlation coefficient (ICC) was calculated to explore consistency within and between examiners for measurements with the original EOS images and the CT-generated DRRs. To evaluate intra-rater reliability, we compared the measurements obtained by two examiners who completed the EOS measurement training and had worked with these measurements for 3 years, and measured all the parameters of the 24 subjects twice with a 1-week interval. To evaluate inter-rater reliability, we compared the measurements obtained by the two examiners of all the parameters of the same 24 subjects in 1 week. An ICC value approaching 1.0 indicates less variability, better consistency, and a value over 0.8 is considered sufficiently reliable.

The values of all the alignment spinopelvic parameters were normally distributed, thus a paired-t-test was performed to compare between supine (CT-generated DRR) and standing (EOS) positions. Type I error (α), power (1-β), and post-hoc sample size for the statistical significance were calculated.

## Results

### Patient demographic data

Mean age and body mass index were 60.1 years (20–80) and 22.2 kg/m^2^ (18.0–31), respectively. Mean value with SD, SE, and 25%/75% interquartile range of all the demographic and radiologic standard parameters are reported in Table 1. The mean ODI score was 31.4% (0–52%) and the mean SRS-22 (subtotal) score was 2.9 (1.8–4.4).

**Table 1:** Demographics of the subject and spinal alignment parameters in standing position measured by EOS (24 women)

	Mean	Range (min/max)	SD	SE	IQ 25%/75%^*1^
Age (years)	60.1	20 / 80	14.2	2.9	59.3 / 67
Body mass index^*2^	22.2	18.0 / 31.0	3.0	0.6	20.0 / 25.0
ODI^*3^ (%)	31.4	0 / 52	12.4	2.5	24.0 / 40.0
SRS-22^*4^	2.9	1.8 / 4.4	0.5	0.1	2.6 / 3.2
VAS^*5^	6.6	0 / 10	2.8	0.6	5.3 / 8.0
T1–12 kyphosis (°)	24.0	−6.8 / 56.2	15.5	3.2	11.1 / 37.6
T4–12 kyphosis (°)	17.8	−10.6 / 45.8	14.5	3.0	9.7 / 25.6
L1-S1 lumbar lordosis (°)	21.8	−32.6 / 63.3	25.5	5.2	5.8 / 44.6
L1-L5 lumbar lordosis (°)	8.5	−38.5 / 66.0	26.1	5.3	−9.6 / 33.5
Sacral slope (°)	27.0	0 / 60.4	14.1	2.9	18.1 / 32.9
Pelvic tilt (°)	30.7	3.8 / 48	10.6	2.2	25.3 / 37.8
Pelvic incidence (°)	57.7	33.6 / 80.8	10.9	2.2	53.0 / 65.0
Cobb’s angle (°) ^*6^	39.5	12.2 / 74.2	20.1	4.7	22.8 / 57.6
Rotation (°) ^*7^	14.7	0.7 / 34.9	10.2	2.4	4.9 / 20.7

^*1^Interquartile range, 25%/75% values

^*2^The body mass index was calculated as the weight in kilograms divided by the square of the height in meters (kg/m^2^)

^*3^The Oswestry Disability Index [12, 13]

^*4^Scoliosis Research Society – 22, subtotal [2, 18]

^*5^Visual analog scale, 0 is no pain and 10 is most severe pain

^*6^Cobb’s angle of the major curve

^*7^Vertebral rotation of the apex in the major curve

### Reliability of the measurements in the supine (CT-generated DRR) and standing (EOS) positions

The mean ICCs of intra-rater reliabilities for supine and standing positions were 0.98 (0.96–1.00) and 0.98 (0.96–1.00), respectively. The mean ICCs of inter-rater reliabilities for supine and standing positions were 0.97 (0.93–0.99) and 0.99 (0.97–1.00), respectively. Overall, the ICC data suggested excellent measurement consistency and reliability in both the standing and supine positions (Table 2).

**Table 2:** Comparison of Intra-Class Correlation Coefficient (ICC) and 95% confidential interval for all the parameters between supine (CT-based DRR) and standing (original EOS)

CT	ICC	95% CI	EOS	ICC	95% CI
Intra-rater reliability (two examiners, 24 subjects)					
T1-T12 TK	0.963	0.905 / 0.986	T1-T12 TK	0.980	0.955 / 0.991
T4-T12 TK	0.967	0.926 / 0.985	T4-T12 TK	0.989	0.976 / 0.995
L1-L5 LL	0.979	0.953 / 0.991	L1-L5 LL	0.986	0.968 / 0.994
L1-S1 LL	0.992	0.981 / 0.996	L1-S1 LL	0.991	0.980 / 0.996
PI	0.982	0.959 / 0.992	PI	0.981	0.956 / 0.992
SS	0.990	0.976 / 0.995	SS	0.984	0.964 / 0.993
PT	0.996	0.992 / 0.998	PT	0.998	0.996 / 0.999
Cobb angle	0.989	0.972 / 0.996	Cobb angle	0.991	0.976 / 0.996
Rotation	0.973	0.930 / 0.990	Rotation	0.955	0.887 / 0.983
Average	0.981	0.955 / 0.992	average	0.984	0.962 / 0.993
Inter-rater reliability (two examiners, 24 subjects)					
T1-T12 TK	0.932	0.850 / 0.970	T1-T12 TK	0.988	0.972 / 0.995
T4-T12 TK	0.971	0.935 / 0.987	T4-T12 TK	0.984	0.964 / 0.993
L1-L5 LL	0.964	0.919 / 0.984	L1-L5 LL	0.989	0.974 / 0.995
L1-S1 LL	0.986	0.963 / 0.994	L1-S1 LL	0.993	0.976 / 0.997
PI	0.969	0.821 / 0.990	PI	0.974	0.940 / 0.988
SS	0.964	0.466 / 0.991	SS	0.982	0.945 / 0.993
PT	0.992	0.846 / 0.998	PT	0.997	0.917 / 0.999
Cobb angle	0.992	0.928 / 0.998	Cobb angle	0.994	0.985 / 0.998
Rotation	0.963	0.905 / 0.986	Rotation	0.974	0.931 / 0.990
Average	0.970	0.848 / 0.989	average	0.986	0.956 / 0.994

### Comparison of spinal and pelvic parameters between the supine and standing positions

**Fig. 3:**
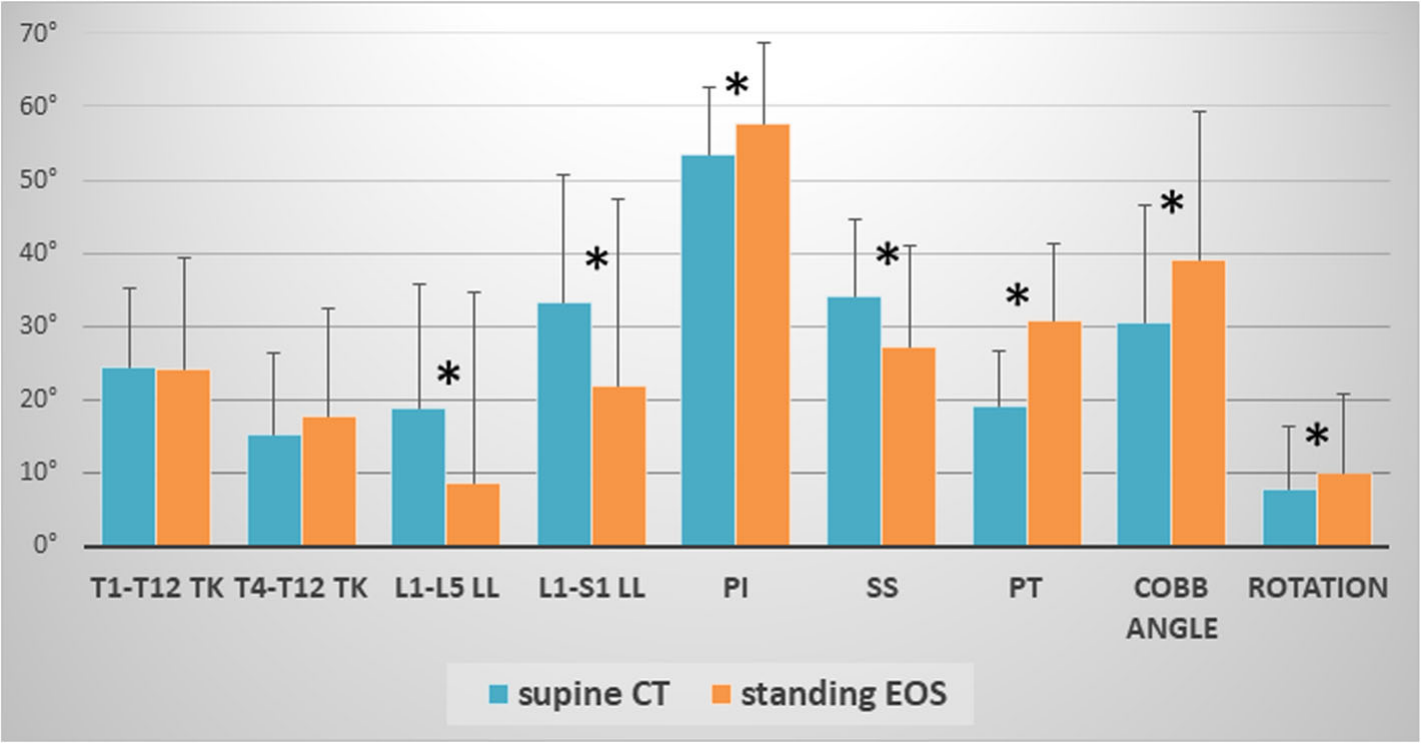
Mean (+ standard deviation) values of spinopelvic parameters measured in supine (blue bars) and standing (orange bars) positions Asterisks denote statistically significant differences (*p* < 0.05) based on paired t-test

Thoracic kyphosis in the standing position was not different from that in the supine position (Table 3, Fig. 3). On the other hand, LL at both in L1-L5 and L1-S1 levels was significantly smaller in the standing position than in the supine position. This means that the lumbar spine is more kyphotic in the standing position than in the supine position.

**Table 3:** Comparison of the values of standard parameters between supine (CT-generated DRR) and standing (original EOS) positions by paired t-test

Parameters (°)	position	Image modality	Mean	Range (min/max)	SD	SE	95% CI^*1^	Type I error (*α*)
T1-T12 kyphosis	supine	CT	24.4	−2.2 / 44.7	10.9	2.2	19.8 / 29.0	*0.8581*
	standing	EOS	24.0	−6.8 / 56.2	15.5	3.2	17.5 / 30.5	
T4-T12 kyphosis	supine	CT	15.3	−10.3 / 35.7	11.1	2.3	10.7 / 20.0	*0.1641*
	standing	EOS	17.8	−10.6 / 45.8	14.5	3.0	11.7 / 24.0	
L1-S1 LL^*2^	supine	CT	**33.1**	−11 / 55.9	17.5	3.8	25.7 / 40.5	***0.0002***
	standing	EOS	**21.8**	−32.6 / 63.3	25.5	5.2	11.0 / 32.6	
L1-L5 LL	supine	CT	**18.9**	−20.3 / 45.1	16.9	3.5	11.7 / 26.0	***0.0010***
	standing	EOS	**8.5**	−38.5 / 66.0	26.1	5.3	−2.5 / 19.5	
SS^*3^	supine	CT	**34.1**	11.1 / 48.5	10.5	2.2	29.7 / 38.6	***0.0003***
	standing	EOS	**27.0**	0 / 60.4	14.1	2.9	21.1 / 33.0	
PT^*4^	supine	CT	**19.2**	5.2 / 37	7.5	1.5	16.1 / 22.4	***< 0.0001***
	standing	EOS	**30.7**	3.8 / 48	10.6	2.2	26.2 / 35.2	
PI^*5^	supine	CT	**53.4**	32.2 / 68.1	9.2	1.9	49.5 / 57.3	***0.0013***
	standing	EOS	**57.7**	33.6 / 80.8	10.9	2.2	53.1 / 62.3	
Cobb angle^*6^	supine	CT	**31.0**	6.8 / 57	15.3	3.6	23.4 / 38.7	***0.0001***
	standing	EOS	**39.5**	12.2 / 74.2	20.1	4.7	29.5 / 49.4	
Rotation^*7^	supine	CT	**11.7**	0.1 / 25.0	7.6	1.8	8.0 / 15.5	***0.0318***
	standing	EOS	**14.7**	0.7 / 34.9	10.2	2.4	9.7 / 19.8	

^*1^95% confidence interval

^*2^Lumbar lordosis

^*3^Sacral slope

^*4^Pelvic tilt

^*5^Pelvic incidence

^*6^Cobb’s angle of major curve (°)

^*7^Vertebral rotation of the apex in the major curve (°)

Mean values with bold letters indicate statistically significance (*p* < 0.05) between supine (CT) and standing (EOS)

With regard to pelvic alignment, SS was significantly smaller in the standing position than in the supine position. In contrast to the SS value, PT was significantly greater in the standing position than in the supine position. PI was significantly greater in the standing position than in the supine position (Table 3, Fig. 3).

The Cobb and rotation angles of the major curve were significantly greater in the standing position than in the supine position. Statistical power was at least 0.8 in all parameters. Post hoc calculated sample size necessary for sufficient power was less than 24 for all parameters except for T1-T12 / T4-T12 kyphosis and Rotation.

## Discussion

Adults with spinal deformities generally have a lower health-related quality of life. The present findings are consistent with findings of previous studies in that the patients in our population had poor low-back pain-related quality of life. Fairbank et al. [12] reported that the mean ODI score in a normal Caucasian population is 10.2. In addition, Tonosu et al. [27] reported that the mean ODI in a normal Japanese population is 8.73 (8.80 in men, 8.66 in women) when corrected for age, and a tendency for the ODI to gradually increase with advancing age. The mean ODI score in the present study (31.4%) was much greater than these values and mean SRS-22 score in our study (2.9) was lower than the normal value [5]. To clarify the pathology leading to the lower quality of life in patients with ASD, a precise comparison of supine and standing measurements using the same modality and measurement method is needed.

The difference in curve magnitude between standing and supine positions was studied previously in AIS patients. Keenan et al. [20] investigated the difference in the Cobb angle between the supine position (reconstructed CT) and standing position (conventional X-ray) in 52 patients with AIS having a mean age of 14.6 years. They reported a mean Cobb angle on standing radiographs of 51.9°, which was a significantly greater value than the mean Cobb angle on supine CT images of 40.5° [20]. The Cobb angle measurement, however, includes a fundamental error related to the selection of the vertebral endplates. In another study [24], spinal parameters were measured on 50 anteroposterior radiographs of scoliotic spines, on 6 separate occasions each by 4 orthopedic surgeons using the Cobb method; they reported that the 95% confidence limit for intraobserver variability was 4.9° and that for interobserver variability was 7.2°. Thus, due to the inherent inter- and intra-observer error in conventional X-ray measurements, it is difficult to conclude whether there are quantitative differences in the curve magnitude due to the positioning of the subjects.

In the present study, we transformed the CT dataset (supine position images) into an EOS-like dataset with the DRR technique, which generates two-dimensional X-ray-like images using the same calibration parameters and geometry as the EOS cabin. The projected anteroposterior and lateral DRRs were used as inputs for routine-like EOS stereoradiographic spine modeling with sterEOS software. The EOS data (standing position) and the CT-generated DRRs (supine position) were then compared. In the present study, we confirmed the reliability of this comparison method using repeatability tests with ICC in the 24 subjects, and found that the ICCs were excellent in all the values, both in CT-generated DRRs and EOS measurements. Thus, the inherent error due to measurement and different imaging modalities was overcome by the comparison with the EOS 3D measurement system.

In adult-to-elderly patients with spinal deformity, sagittal malalignment is more important than coronal alignment [14]. In contrast to AIS, adult-to-elderly patients with spinal deformity complain that they tend to stoop with back pain or radicular pain of the lower extremities, and the symptoms disappear when they lie down. Therefore, the positional change of the 3D whole spine alignment should be clarified, especially in aging adults. In the present study with patients having a mean age of 60.1 years, the Cobb angle and Rotation angle of the major curve, mostly the thoracolumbar area, were significantly greater in the standing position than in the supine position. Lumbar lordosis in the standing position was significantly more kyphotic and the pelvis was significantly more retroverted, with a smaller SS and greater PT, compared with that in the supine position. The axial skeleton of human beings is aligned as a chain of balance in the standing position with the “cone of economy” in healthy subjects [8]. The standing full-body sagittal alignment and balance in reference to the gravity line were recently described in a healthy adult population using EOS radiographs [17]. The results of the present study suggest that the “cone of economy” principle deteriorates in patients with spinal deformity, making it difficult to maintain the standing position due to back pain with or without radicular pain.

**Fig. 4:**
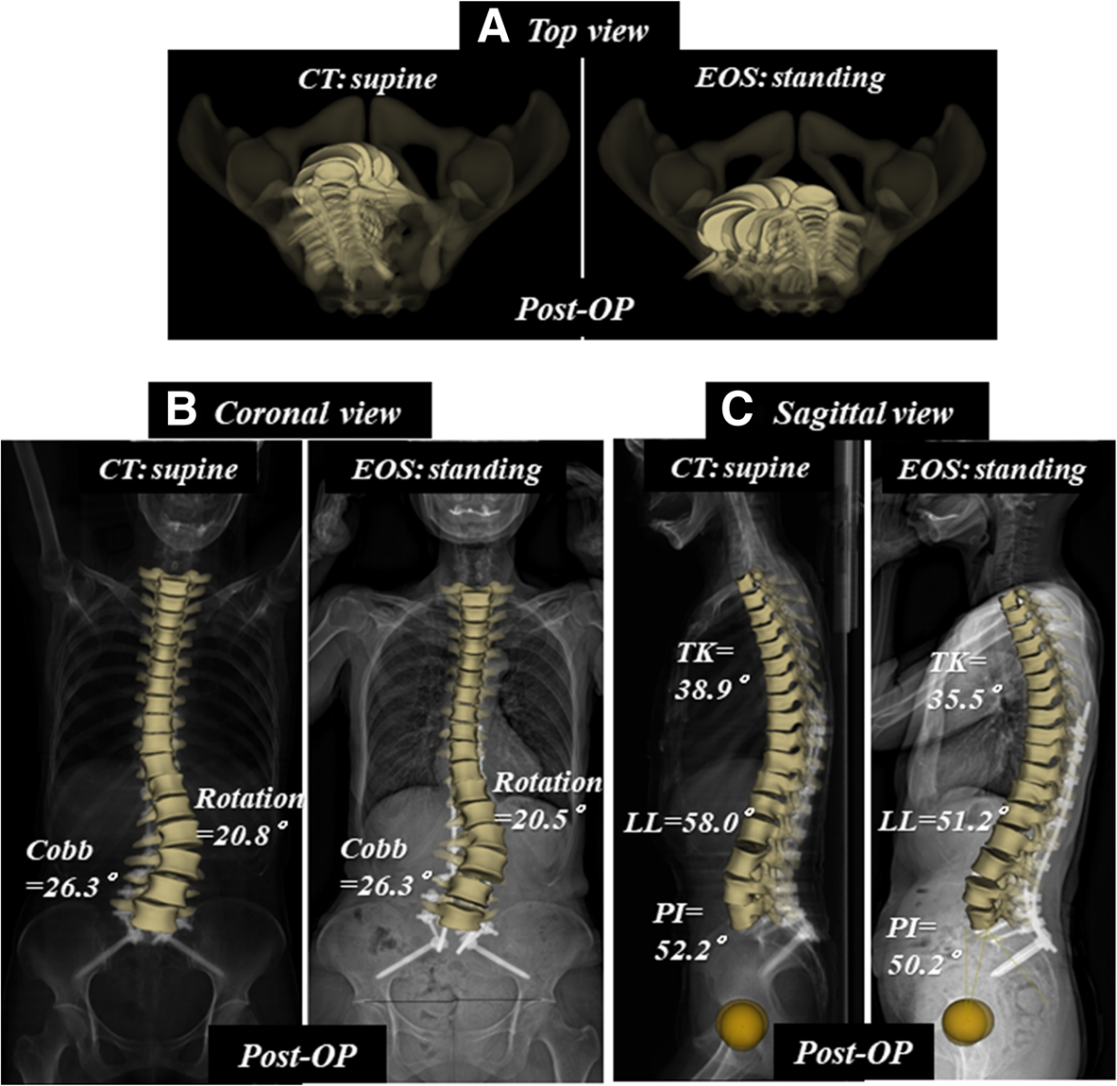
Patient with degenerative kyphoscoliosis (Fig. 2) treated by posterior correction and fusion from T9 to pelvis. Postoperative supine CT and standing EOS images. **a** Top view images. **b** Coronal images. **c** Sagittal images

**Fig. 5:**
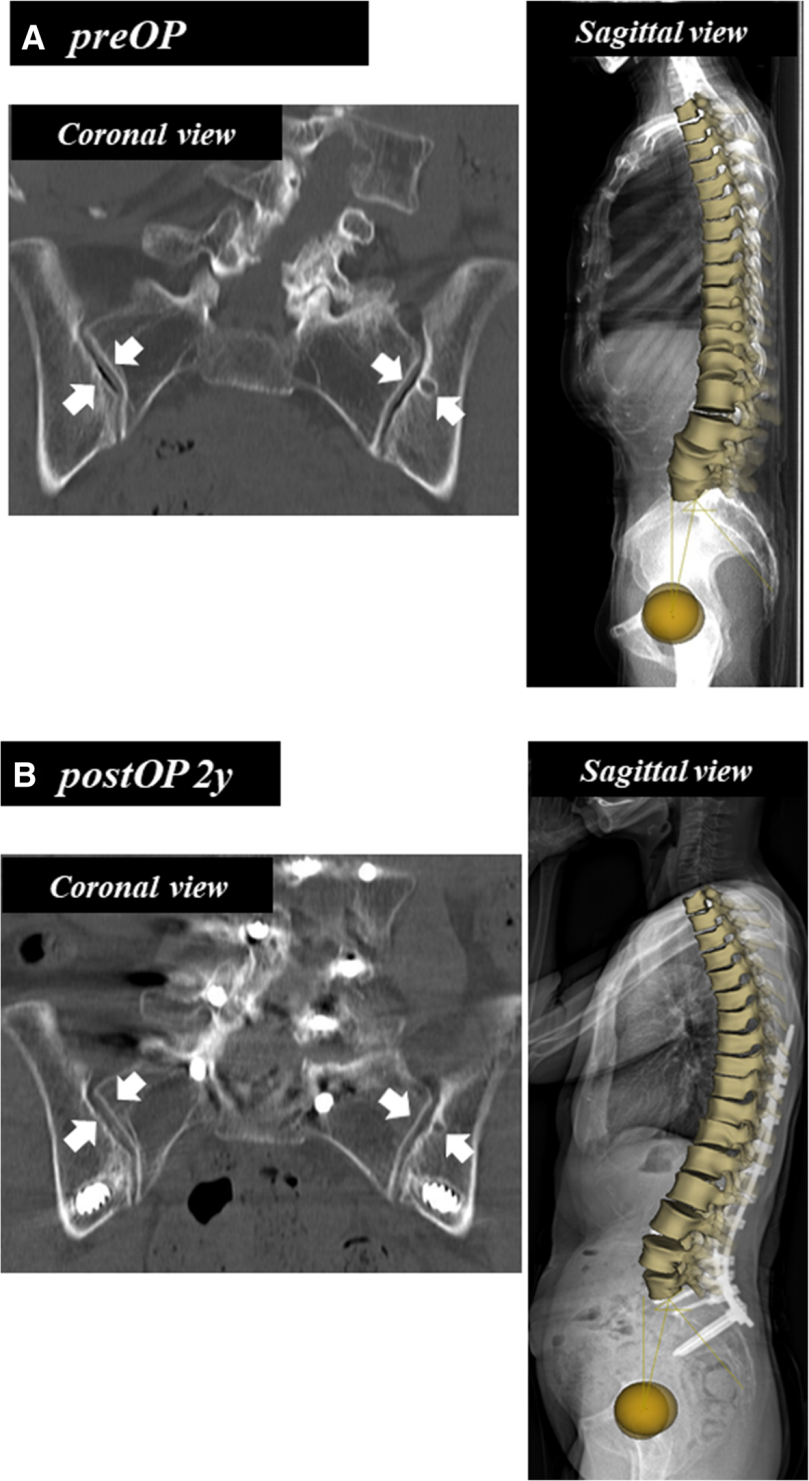
Reconstructive coronal reconstructed CT images of bilateral sacro-iliac joints and corresponding EOS images of the patient (Figs. 2 and 4). **a** Preoperative images. Arrows indicate vacuum in the bilateral sacro-iliac joints and subchondral cyst. **b** Images postoperative 2 years. Arrows indicate vanished vacuum in the joints and diminished subchondral cyst

PI is a fundamental parameter for spinopelvic standing alignment [10, 11, 22], and is believed to be a constant value in each individual. The value was, however, significantly greater in the standing position than in the supine position. Mangion et al. [23] measured PI on radiographs of 30 fetuses, 30 children, and 30 adults, and found that PI considerably increases during the first few months of life, continues to increase during the early years, and stabilizes at around the age of 10 years. In our previous study [16], PI was positively correlated with age, even in healthy adult subjects. PI increased around 10° on average from 20 to 70 years of age, probably due to sacroiliac osteoarthritis [16]. This finding is compatible with a previous review article on sagittal pelvic alignment parameters, which described that PI tends to increase with age in both normal and scoliotic subjects [29]. In the present study, we demonstrated a significant difference in PI between the supine and standing positions. The reconstructed coronal CT image of a case with degenerative kyphoscoliosis (Fig. 4a) shows osteoarthritis of the sacroiliac joint with a vacuum phenomenon, subchondral osteosclerosis, and cyst formation (Fig. 5a). The preoperative PI was 57.1° in standing EOS (49.8° in supine CT) and the PI decreased to 50.2° in standing EOS (52.2° in supine CT) following spinopelvic correction surgery with bilateral S2 alar-iliac fixations (Fig. 4b) [25]. Furthermore, the vacuum in the sacroiliac joint disappeared and the cyst became smaller after surgery as a result of fixation by the screws stabilizing the sacroiliac joints (Fig. 5b). Sacroiliac joints are the only sites that can move between the base of the sacrum and the acetabulum, thereby affecting the PI value. Therefore, our data suggest that the sacroiliac joint moves abnormally due to osteoarthritic changes in ASD cases, leading to changes in the PI between the supine and standing positions. The high ICC for both the supine and standing position values suggests that the difference is a true difference, and not due to measurement variability. PI is considered a set characteristic of the individual spinopelvic shape, but it can change due to degenerative processes of the spine. Thus, it is important to remember that the aging and degenerative processes affect the whole spinopelvic alignment and balance in reference to patient positioning.

The findings in the present study offer a basic pathology of ASD with positional change in thoraco-lumbar to pelvic alignment, suggesting that a main focus of the correction and fusion surgery is the lumbar to pelvic area and further extension of the fusion to upper thoracic area is not necessary. Furthermore, we believe that the results contribute not only for spine surgeons but also for the professionals involved in the conservative management of patients with back pain and spine deformities by keeping in mind the sagittal spinopelvic profile in the supine position which could represent a starting point. Potential limitations of this study are, however, the wide age range of the patients but relatively small sample size. Aging and spondylotic change affect the spino-pelvic flexibility. Thus, although the results in the present study is statistically accurate, we need to continue the investigation with further sample size.

## Conclusion

We established a method for comparing spinopelvic alignment between the supine and standing positions by converting CT DICOM data into an EOS-like dataset, a DRR technique. Comparison revealed that the Cobb angle and axial apical rotation of the major curve, mostly in the thoracolumbar area, were significantly greater in the standing position than in the supine position. Lumbar lordosis in the standing – weight-bearing – position was significantly less lordotic and the pelvis was significantly more retroverted, with a smaller SS, greater PT, and even greater PI, compared with that in the supine position.

## Abbreviations

AIS: Adolescent idiopathic scoliosis
ASD: Adult spinal deformity
Cobb angle: The Cobb angle of the major curve
CT: Computed tomography
DRR: Digitally reconstructed radiograph
ICC: Intra-class correlation coefficient
LL: Lumbar lordosis
MRI: Magnetic resonance image
ODI: Oswestry disability index
PI: Pelvic incidence
PT: Pelvic tilt
Rotation: The apical vertebral rotation in the major curve
SD: Standard deviation
SE: Standard error
SRS-22: Scoliosis Research Society-22 score
SS: Sacral slope
